# Supplementary material for: Extracting user profile via large language models and ontologies
Source: PLoS One. 2026 May 11;21(5):e0329934. doi: 10.1371/journal.pone.0329934 (PMC13160343; doi:10.1371/journal.pone.0329934)
Supplement: S1 File — All supporting figures and tables referenced throughout the text are located in this file. (PDF) [file pone.0329934.s001.pdf]

Supporting information

S1 Table. Performance of our approach and LLMs in each contradiction category in terms of accuracy.

Conflict Category	Ours	Llama	GPT-3.5-turbo	GPT-4	GPT-4o
Geographical Direction vs. City/Province	**75.0**	37.5	12.5	**75.0**	50.0
Country vs. City	77.7	66.6	22.2	55.5	**100**
City vs. Province	**75.0**	12.5	25.0	25.0	25.0
Province vs. Country	**100**	**100**	**100**	**100**	**100**
Gender-based Name vs. Gender	**100**	75.0	75.0	75.0	87.5
Gender vs. Social Titles	**100**	12.5	0	25.0	50.0
Gender vs. Job	**100**	16.6	16.6	16.6	16.6
Age vs. Job	**100**	62.5	37.5	50.0	87.5
Age vs. Degree	**100**	50.0	37.5	25.0	62.5
Job vs. Degree	**87.5**	37.5	25.0	50.0	62.5
Parenthood vs. Marital Status	**100**	**100**	87.5	87.5	**100**

```
Based on the speaker utterance and the previous system utterance in a conversation, answer these questions as briefly as possible. If the answer to a question is not in the utterance, say "I don't know".

Questions:
1. How many siblings does the speaker have?
2. Is the speaker male or female?
3. How old is the speaker?
4. What is the speaker's job?
5. What is the speaker's hobby?
6. How many sons and daughters does the speaker have?
7. What is the section, city, province, or country where the speaker lives?
8. Is the speaker single or married?
9. What is the speaker's name?
10. What is/are the main topic(s) of this utterance in order? (The topic can be one of these: "marital status", "name", "place of residence", "hobby", "job", "gender", "family", "education", "weather", "goodbye", "greeting", and "other")

Previous system utterance: [system utterance]
Speaker utterance: [speaker utterance]
```

S1 Fig. SF & ID prompt used in zero-shot scenario in the first step.

```
Based on the user's input and the previous chatbot utterance in a Persian conversation, answer the following questions in the shortest possible form in Persian. If the answer is not explicitly or nearly explicitly mentioned in the utterance, say "I don't know".

Questions:
1.  Has the user explicitly or nearly explicitly mentioned how many siblings they have? If yes, write in the form of "#No brother", "#No sister" and "#No sibling". If not, say "I don't know".
2.  Has the user explicitly or nearly explicitly mentioned their gender? If yes, specify it just with “Male“ or “Female”. If not, say "I don't know". (do not imply gender from name)
3.  Has the user explicitly or nearly explicitly mentioned their age? If yes, what is it? (if birthdate with year was mentioned, just write year) If not, say "I don't know".
4.  Has the user explicitly or nearly explicitly mentioned their job? If yes, what is it? If not, say "I don't know". If there are multiple jobs mentioned, separate them with ','.
5.  Has the user explicitly or nearly explicitly mentioned a hobby (an activity they can do in their leisure time)? If yes, what is it? If not, say "I don't know". If there are multiple hobbies mentioned, separate them with ','.
6.  Has the user explicitly or nearly explicitly mentioned how many sons or daughters they have? If yes, write in the form of "#No daughter", "#No sons" and "#No children". If not, say "I don't know".
7. Has the user explicitly or nearly explicitly mentioned their residence (district, city, province, or country)? If yes, what is it? If not, say "I don't know".
8. Has the user explicitly or nearly explicitly mentioned if they are single or married? If yes, specify it just with “Married” or “Single”. If not, say "I don't know".
9. Has the user explicitly or nearly explicitly mentioned their name? If yes, what is it? If not, say "I don't know". If there are multiple names mentioned, separate them with ','.
10. What is/are the main topic(s) in the user's utterance in order? If there are multiple main topics, separate them with ','. (The topic can only be one of these: "marital status", "name", "place of residence", "hobby", "job", "gender", "family", "education", "weather", "goodbye“ and "greeting“. If it is outside of these, just answer "other")

**Note**: Distinguish clearly between the user's workplace and residence. Only consider information that is explicitly mentioned in the user's input and avoid making assumptions. If the information is not available, respond with "I don't know".
**Note**: About question 1, if the user uses phrases like “من تک بچه ام”, “من تک فرزندم” or “من تنها فرزند خانواده ام”, understand that they mean the user is an only child and does not have any siblings. These phrases do not indicate anything about the user's children.

Previous chatbot utterance: [chatbot utterance]
User’s input: [user utterance]
```

S2 Fig. Refined SF & ID prompt after investigating LLM results used in zero-shot.

```
Based on the user's input and the previous chatbot utterance in a Persian conversation, infer and answer the following questions only if the information is explicitly stated or logically inferable from the conversation. If the information is missing or cannot be inferred, response to each question with "I don't know" Responses must be short and precise and if no information is available, always write "I don't know“.
1.What is the total number of siblings the user has? Just write the number or say "I don't know"
2.How many sisters does the user have?  Just write the number or say "I don't know"
3.how many brothers does the user have? Just write the number or say "I don't know"
4.Is the user male or female? Just write "male", "female" or "I don't know"
5.How old is the user? Just write the number or say "I don't know"
6.What is the user's job? Do not explain anything and only write the job title if mentioned or inferable; otherwise, write "I don't know“. If multiple jobs exist, separate them with ','.
7. what is the user's hobby (an activity they can do in their leisure time)? If multiple hobbies exist, separate them with ','.
8. How many children does the user have? Just write the number or say "I don't know“.
9.How many sons does the user have? Just write the number or say "I don't know“.
10.How many daughters does the user have? Just write the number or say "I don't know“.
11.What is the user's residential district/city/province/country? Do not explain anything and only write the user's location if mentioned or inferable; otherwise, write "I don't know“.
12.Is the user married or single? Just write "married", "single" or "I don't know“.
13.What is the user's name? If multiple names exist, separate them with ','.

#Conversation:
chatbot: [chatbot utterance]
user: [user utterance]
```

S3 Fig. Prompt used for assessing LLMs capability in user profile extraction.

```
In the following Persian sentence, identify any probable explicit or implicit semantic contrasts or contradictions. If there is no contrast or contradiction, state that none is present. Provide an explanation for any identified contrast or contradiction.

Sentence: [sentence]
```

S4 Fig. Conflict detection prompt used for zero-shot conflict detection.

```
Consider these sample sentences as negative examples, indicating
the speaker is not a mother:
[list of negative samples]

Consider these sample sentences as positive examples, indicating
the speaker is a mother:
[list of positive samples]

Now, given the input sentence: [sentence]

Does this sentence imply that the speaker is a mother? Just answer
with "Yes" or "No".
```

S5 Fig. Prompt employed for LLM-based inference engine.
